# Prediction, perception and agency

**DOI:** 10.1016/j.ijpsycho.2011.11.014

**Published:** 2012-02

**Authors:** Karl Friston

**Affiliations:** The Wellcome Trust Centre for Neuroimaging, Institute of Neurology, University College London, 12 Queen Square, London WC1N 3BG, UK

**Keywords:** Predictive coding, Perception, Agency, Free energy, Inference, Schizophrenia

## Abstract

The articles in this special issue provide a rich and thoughtful perspective on the brain as an inference machine. They illuminate key aspects of the internal or generative models the brain might use for perception. Furthermore, they explore the implications for a sense of agency and the nature of false inference in neuropsychiatric syndromes. In this review, I try to gather together some of the themes that emerge in this special issue and use them to illustrate how far one can take the notion of predictive coding in understanding behaviour and agency.

## 1 Introduction

Having read the articles comprising this special issue, I was struck by how coherent they were and how they provide a compelling narrative about how the brain responds to its sensory impressions, the implications for subsequent action and the genesis of (and failures of) a sense of agency. This review tries to summarise this narrative, in theoretical terms, using the main points made by the individual articles.

## 2 Perception active inference and agency

A common theme that runs throughout this special issue is the notion of the brain as a constructive or predictive organ that actively generates predictions of its sensory inputs using an internal or generative model. This is now a widely accepted view of perception that can be traced back to Helmholtz's original writings on unconscious inference (Helmholtz, 1866/1962). In this review, I want to emphasize that exactly the same principles can also explain action and behaviour; and indeed theory of mind and a sense of agency. This may be less apparent for some; in the sense that predictive coding and other formal theories that fit within the inference framework are not generally considered in the context of action and its agency. However, perception and action can be related formally, through a common minimization of prediction error (Friston et al., 2011).

This is trivially true, in the sense that under predictive coding, perception is the job of optimising predictions to minimize sensory prediction error, while movement invariably suppresses proprioceptive (e.g., stretch receptor) prediction errors at the level of the spinal cord and cranial nuclei. This is nothing more than equipping a predictive coding scheme with classical motor reflex arcs. At a more theoretical level, one can use ergodic arguments about the nature of self organising systems to reach exactly the same endpoint; namely, an imperative to minimize prediction errors or surprise through action. In this view, perception can be regarded as providing veridical predictions about both exteroceptive (e.g., visual) and proprioceptive (e.g., kinaesthetic) sensations that enable action or motor behaviour to sample sensory inputs selectively to ensure predictions come true. This is an important observation because it speaks to a substantial proportion of the articles in this special issue that deal not just with the auditory or visual consequences of a stimulus but their behavioural and, implicitly, proprioceptive consequences. At a more abstract level, predictions about how we will physically move are composed and generated in a way that determines how we behave. Perhaps the most important determinants of our behaviour (and their underlying predictions) are beliefs about the intentions and behaviour of others. This necessarily requires an internal model of self in relation to others and an implicit sense of agency. The intimate relationship between veridical and adaptive predictions, subsequent action and a sense of agency is illustrated very nicely by the succession of themes covered in this special issue. In what follows, we will consider hierarchical perceptual inference in the auditory and visual domains, active inference in terms of behaviour and responses, how this pertains to a sense of agency and, finally, failures of inference in psychopathology.

## 3 Hierarchical perceptual inference

It is evident that there is a consensus about the nature of predictions and their relationship to generative models in the Helmholtzian tradition. This is articulated very nicely by the two papers co-authored by the special issue's guest editors. For example, Bendixen et al. (Bendixen et al., 2012-this issue) start from the psychological perspective of mental models which “simulate our reality”. They highlight the connections with the “theory of unconscious inference of Herman von Helmholtz” and how these ideas have been progressed to accommodate “the proactive or predictive aspect of mental models” in perception. Their emphasis is on a “truly predictive account of auditory processing (as opposed to a retrospective verification of predictions)”. The mediator of this predictive optimisation is clearly prediction error; namely “signals of violated predictions (mismatch signals such as a mismatch negativity and stimulus omission responses)”. The same theme is taken up by Winkler and Czigler (2012-this issue), who provide a concise and compelling summary:

*Helmholtz's notion of unconscious inference engendered arguably the most fruitful line of perceptual research throughout the relatively short history of psychology, the empiricist tradition. In one of its contemporary variants, Gregory suggests that a percept is akin to a scientific hypothesis; it is the brain's best fitting model for the information entering the senses. But together with Gordon, we can ask how these models are formed, what evidence they are tested against and how do they adapt to an ever changing environment? To answer these questions, some of the theories of predictive coding invoke the principle of free energy minimisation.*

Free energy is a quantity from information theory that quantifies the amount of prediction error or, more formally, it is a variational approximation to the surprise or negative log likelihood of some data given an internal model of those data (Friston, 2010). A particular and neurobiologically plausible example of variational free energy minimisation is *predictive coding*: “Predictive coding theories posit that the perceptual system is structured as a hierarchically organised set of generative models with increasingly general models at high levels” (Winkler and Czigler, 2012-this issue). The notion of *hierarchies* is essential to perceptual inference and many of the issues addressed below. Mathematically, hierarchical generative models enable the brain to optimize its own prior beliefs. In statistics, these beliefs are known as *empirical priors* and endow models with rich or deep structure. This is illustrated nicely in the contribution of van Petten and Luka (2012-this issue), who relate various components of the event related potential (ERP) to the confirmation or disconfirmation of predictions; in other words, the elaboration of prediction errors. Crucially, they show that late responses “consist of two distinct components with different scalp topographies”, one associated with semantically incongruent words and one associated with congruent words. This illustrates the hierarchical nature of generative models that the brain might employ, in the sense that congruence provides a hierarchical context for semantics. From the point of view of hierarchical models, this context would normally be considered a higher level attribute of the models generating predictions. The very fact that van Petten and Luka can demonstrate the electrophysiological correlates of contextual effects suggests that the brain must indeed be representing hierarchical attributes of the sensorium and may be so doing in terms of prediction errors. I also liked their observation that the meaning of “predict” derives from its origin in Latin — *pre* (before or in front of) plus *dicere* (to speak). In other words, “to declare what will happen in the future”.

**Fig. 1:**
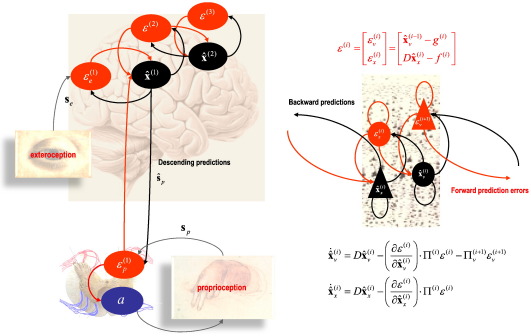
This figure illustrates the neuronal architectures that might implement predictive coding and active inference. The left panel shows a schematic of predictive coding schemes in which Bayesian filtering is implemented by neuronal message passing between superficial (red) and deep (black) pyramidal cells encoding prediction errors and conditional predictions or estimates respectively (Mumford 1992). In these predictive coding schemes, top-down predictions conveyed by backward connections are compared with conditional expectations at the lower level to form a prediction error. This prediction error is then passed forward to update the expectations in a Bayes-optimal fashion. In active inference, this scheme is extended to include classical reflex arcs, where proprioceptive prediction errors drive action — *a* (alpha motor neurons in the ventral horn of the spinal-cord) to elicit extrafusal muscle contractions and changes in primary sensory afferents from muscle spindles. These suppress prediction errors encoded by Renshaw cells. The right panel presents a schematic of units encoding conditional expectations and prediction errors at some arbitrary level in a cortical hierarchy. In this example, there is a distinction between hidden states **x**_*x*_ that model dynamics and hidden causes **x**_*v*_ that mediate the influence of one level on the level below. The equations correspond to a generalized Bayesian filtering or predictive coding in generalized coordinates of motion as described in (Friston, 2010). In this hierarchical form *f*^(*i*)^ : = *f*(**x**_*x*_^(*i*)^, **x**_*v*_^(*i*)^) corresponds to the equations of motion at the *i*-th level, while *g*^(*i*)^ : = *g*(**x**_*x*_^(*i*)^, **x**_*v*_^(*i*)^) link levels. These equations constitute the agent's prior beliefs. *D* is a derivative operator and Π^(*i*)^ represents precision or inverse variance. These equations were used in the simulations presented in the next figure.

The fundamental architectural role of hierarchies in cross modal sensory integration suggests that *amodal* concepts at higher levels in a hierarchy should generate *multimodal* predictions and associated prediction errors. This rather obvious observation fits comfortably with the formal similarities between mismatch or prediction error responses in the auditory domain and equivalent responses in the visual domain. Indeed, Cheung and Bar (2012-this issue) provide a compelling analysis of prediction and generative modelling from the point of view of visual research in cognitive neuroscience. The mechanistic implications of the visual mismatch negativity are explored in Kimura (2012-this issue) who lends this interpretation a validity, in terms of things such as representational momentum and perceptual sequence learning. Crucially, Kimura invokes “a bi-directional cortical network that includes the visual extra striate in prefrontal areas”:

The notion of bi-directional message passing in cortical networks is a key tenet of predictive coding. For those readers not familiar with neuronal implementations of predictive coding, it is remarkably simple: Current thinking is that the representations of the causes of sensory inputs (for example an object in the field of view or an auditory object) are encoded at multiple hierarchical levels by the activity of deep pyramidal cells that send backward projections to lower cortical levels. These backward projections preferentially target superficial layers in the cortex that house superficial pyramidal cells thought to report prediction errors. Prediction errors are simply the difference between the representations encoded at any level in the hierarchy and the top-down predictions generated by the brain's internal model. The formation of prediction errors rests upon simple synaptic mechanisms, where the predictions inhibit prediction error units (polysynaptically), while the representational units being predicted excite them. The resulting activity in superficial pyramidal cells is then passed through forward extrinsic connections to adjust representational units at higher levels. This reciprocal message passing proceeds until prediction error is minimised throughout all levels of the hierarchy; thereby affording a Bayes-optimal representation of sensory causes at multiple levels of description. Fig. 1 illustrates this scheme.

Interestingly, given the focus of this special issue on event related potentials, the superficial pyramidal cells thought to report prediction errors are exactly the same cell populations that predominate in non-invasive electromagnetic recordings. In other words, the explanation of a mismatch negativity in terms of prediction error has a high degree of biological plausibility, under these predictive coding schemes. A key aspect of hierarchical representations or generative models is exemplified in the papers on music perception. As noted by Rohrmeier and Koelsch (2012-this issue), “It is well understood that constant predictive activity is indispensable and vital for survival”. This constancy speaks to multiple temporal scales and a separation of those scales in a hierarchical setting. This is illustrated nicely in Schwartze et al. (2012-this issue) who describe “a model of temporal processing in audition and speech that involves a division of labour between the cerebellum and the basal ganglia in tracing acoustic events in time”. In their model, they assign the cerebellum a special role in modelling temporal structure with a high temporal precision, while the basal ganglia-thalamo-cortical system evaluates slower “longer range” temporal structure. This “division of labour” is exactly the same in formal models of predictive coding. In these models, high level central pattern generators (that exhibit winnerless competition) provide the slower temporal structure that guides faster dynamics controlling the amplitude and frequency modulation of predicted acoustic input (Kiebel et al., 2009). Although “fully fledged music prediction cannot be modelled at present” (Rohrmeier and Koelsch, 2012-this issue) both simulations of this sort and the empirical evidence that they review, shed light on the nature of “interactive predictive mechanisms”.

Up until now, we have focussed on the minimisation of prediction error in terms of self organised neuronal activity in response to sensory surprises or new stimuli. However, exactly the same principle can be applied to any other attribute of the brain's generative model; including synaptic connection strengths or efficacy. The suppression of prediction errors through changes in coupling among brain areas corresponds to *perceptual learning* and can be expressed in a form that is very similar to classical associative or Hebbian plasticity (Friston, 2010). Janacsek and Nemeth look at perceptual learning in terms of consolidation and, in particular “implicit sequence learning that not only underlies motor but cognitive and social skills as well”. Interestingly, they note consolidation of memory traces after the initial acquisition can “result in increased resistance to interference or even improvement in performance following an offline period”. This is a fascinating observation that suggests optimisation of the brain's generative model does not necessarily need online sensory data. Indeed, there are current theories about the role of sleep in optimising the brain's generative model, not in terms of its ability to accurately predict data, but in terms of minimising complexity. Mathematically, this is interesting because surprise or model evidence can be decomposed into accuracy and complexity terms; suggesting that model evidence can be increased by removing redundant model components or parameters (Friston, 2010). This provides a nice Bayesian perspective on synaptic pruning and the issues considered by Németh and Janacsek (2012-this issue).

## 4 Active inference

As noted above, a simple extension to predictive coding is to consider their suppression by the motor system. In this extension, prediction errors are not just suppressed by optimising top-down or descending predictions but can also be reduced by changing sensory input. This does not necessarily mean visual or auditory input but the proprioceptive input responding to bodily movements. As noted above, the suppression of proprioceptive prediction errors is, of course, just the classical reflex arc. In this view, motor control becomes a function of descending predictions about anticipated or predicted kinematic trajectories. See Fig. 1 for a schematic. The important observation here is that the same sorts of synaptic mechanisms and inferential principles can be applied to both perception and the consequences of action. This nicely accommodates the literature on error related negativity reviewed by Hoffmann and Falkenstein (2012-this issue); who consider the “monitoring of one’s own actions” and its role in adjusting behaviour. Again, the focus is on EEG, suggesting that even within single trial recordings, the neurophysiological correlates of behaviour-dependent prediction errors can be observed empirically. In their words: “The initiated response is compared with the desired response and a difference; i.e., mismatch between both representations induces the error negativity”. This is not the proprioceptive prediction error that drives reflex arcs but a high level perceptual (or indeed conceptual) prediction error; suggesting that the long-term hierarchical predictions of unfolding sensory and kinematic changes have been violated. In other words, these phenomena speak again to separation of temporal scales and hierarchies in providing multimodal predictions to the peripheral sensory and motor systems.

Active inference means that movements are caused by top-down predictions, which means that the brain must have a model of what caused these movements. This begs the interesting question as to whether there is any sense of agency associated with representations. In other words, if I expect to move my fingers and classical motor reflexes cause them to move, do I need to know that it was me who initiated the movement? Furthermore, can I disambiguate between me as the agent or another. These are deep questions and move us on to issues of self modelling and action observation:

## 5 Action observation and agency

**Fig. 2:**
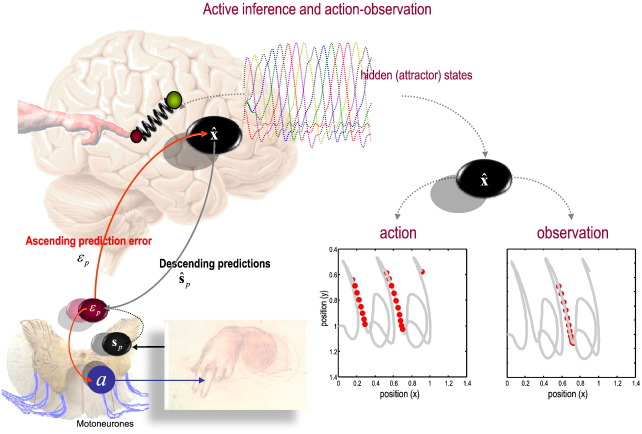
This schematic summarizes the results of the simulations of action observation reported in (Friston et al., 2011). The left panel pictures the brain as a forward or generative model of itinerant movement trajectories (based on winnerless competition, whose states are shown as a function of time in coloured lines). This model furnishes predictions about visual and proprioceptive inputs, which prescribe movement through reflex arcs at the level of the spinal cord (insert on the lower left). The variables have the same meaning as in the previous figure. These predictions include the Newtonian mechanics of a two jointed arm, whose extremity (red ball) is drawn to a target location (green ball) by an imaginary spring. The location of the target is prescribed (in an extrinsic frame of reference) by the high-level winnerless competition. These dynamics and the mapping to an extrinsic (movement) frame of reference constitute the agent's prior beliefs. The ensuing posterior beliefs are entrained by visual and proprioceptive sensations by prediction errors during the process of inference, as summarized in the previous figure. The resulting sequence of movements was configured to resemble handwriting and is shown as a function of location over time on the lower right (as thick grey lines). The red dots on these trajectories signify when a particular neuron or neuronal population encoding one of the hidden states was active during *action* (left panel) and *observation* of the same action (right panel): More precisely, the dots indicate when responses exceeded half the maximum activity and are shown as a function of limb position. The left panel shows the responses during action and illustrates both a place-cell like selectivity and directional selectivity for movement in an extrinsic frame of reference. The equivalent results on the right were obtained by presenting the same visual information to the agent but removing proprioceptive sensations. This can be considered as a simulation of action observation and mirror neuron like activity.

In a nice analysis of agency, gait and self consciousness, Kannape and Blanke (2012-this issue) start by acknowledging: “Agency is an important aspect of bodily self consciousness, allowing us to separate own movements from those induced by the environment and to distinguish own movements from those of other agents”. They also note that “participants self attribute their own movements (i.e. experience a sense of agency for their own movements)”, even when exogenous perturbations cause them to unconsciously change their movement trajectories. These observations tell us something extremely subtle but profound about the representation of self and the production of behaviour: We are apparently remarkably ignorant of perturbations and fluctuations to our intended movements; ignorant in the sense that they do not need to be represented explicitly. This fits comfortably within the predictive coding framework, in that if low-level (reflex arc) mechanisms can explain away prediction error in the periphery, then there is no need to adjust perceptual representations to accommodate them centrally. This suggests that movements are indeed the product of top-down predictions, whose violations are not detected *post hoc* but are eliminated at all levels of the hierarchy, including the spinal cord. Formal simulations of predictive coding have shown their remarkable robustness to exogenous perturbations and also provide a nice metaphor for action observation and the shared representation of perceptual inference about the movements of self and others (see Fig. 2). The example in Fig. 2 starts to address the notion of models of self and others that guide behavioural sequences, perhaps in language and social discourse. Gomot (2012-this issue) consider autism spectrum disorder (ASD) in terms of a failure of implicit high level generative models of others and the interpersonal context in which motor behaviour is generated (predicted). Indeed they note, “substantial differences in how the ASD predicts the environment might have a fundamental role in the deficit revealed in the highly unpredictable social world”. They develop their argument in terms of a compromised ability to build “flexible” predictions and discuss the putative role of impaired top-down influences. It is precisely these top-down or descending influences that predictive coding will associate with predictions and, at the sensorimotor level, constitute the motoric substrates of interpersonal communication and avoidance.

The basic message here is that a fundamental failing of predictive coding mechanisms may underpin many neuropsychiatric disorders, particularly those that involve complicated or difficult Bayesian inference problems that predictive coding tries to solve. If this is the case, one might expect empirical evidence for failures of predictive coding at all levels of the hierarchy, including the elaboration of mismatch negativities. This is precisely what we see; and is nicely discussed in the final two papers considered in this review. Todd et al. (2012-this issue) start by noting that “since the first publication in 1991, over 120 papers have commented on the reduced amplitude of mismatch negativity in schizophrenia”. They pursue the interpretation of the MMN as “a prediction error signal” and link the theoretical neurobiology inherent in predictive coding with the pathophysiology and psychopathology of schizophrenia. It is interesting that, from a theoretical perspective, one of the key predictions of abnormal inference in predictive coding would be an abnormal perceptual learning and reduced amplitude of prediction error signals in the odd-ball paradigm. At the same time, empirical studies over the past decade or so have highlighted this as probably one of the most consistent electrophysiological signatures of schizophrenia. Ford and Mathalon (2012-this issue) take up the theme of predictive coding and schizophrenia explicitly, in terms of a sense of agency. Their focus here is on the efference copy and *corollary discharge* systems that, in classical motor control, are copies of motor commands that enable the brain to accommodate the consequences of its own action. They report studies that show “that auditory cortical responses to speech sounds during talking are reduced compared to when they are played back”. Furthermore, this suppression is reduced in schizophrenia. These results tie together predictive coding, active inference, action observation, agency and psychopathology in a beautiful way: using the language above, efference copy (or more specifically corollary discharge) corresponds to top-down predictions of the auditory consequences of articulatory movement. In other words, one has in mind a high level representation of a speech act that generates bimodal (exteroceptive or auditory and proprioceptive or kinematic) predictions. The former correspond to corollary discharge and the latter to descending proprioceptive drive to the cranial nuclei responsible for speech. The auditory consequences of articulating a word are thereby predicted by the corollary discharge, so as to reduce prediction error. Clearly, this reduction is not available when simply listening to spoken words — a phenomena exploited by Ford and Mathalon. The fascinating issues here are the consequences of breaking this system and the implications for attributing the agency of perceived speech to self or others; and its role in hallucinations and other positive systems of schizophrenia.

## 6 Conclusion

In conclusion, we have seen how many papers in this special issue can be read like the chapters of a book that takes us from the basic fundaments of perceptual inference to a plausible and principled understanding of auditory hallucinations in schizophrenia. Clearly, this may reflect the astute way in which authors were invited to contribute but, I suspect, also reflects the fact that when people ask deep questions about how the brain works, they generally this converge on the same veridical answers.
